# Efficacy of the core DNA barcodes in identifying processed and poorly conserved plant materials commonly used in South African traditional medicine

**DOI:** 10.3897/zookeys.365.5730

**Published:** 2013-12-30

**Authors:** Ledile T. Mankga, Kowiyou Yessoufou, Annah M. Moteetee, Barnabas H. Daru, Michelle van der Bank

**Affiliations:** 1 African Centre for DNA Barcoding, Department of Botany and Plant Biotechnology, University of Johannesburg, PO Box 524, Auckland Park 2006, Johannesburg, South Africa

**Keywords:** Core DNA barcodes, medicinal plants, species identification, South Africa

## Abstract

Medicinal plants cover a broad range of taxa, which may be phylogenetically less related but morphologically very similar. Such morphological similarity between species may lead to misidentification and inappropriate use. Also the substitution of a medicinal plant by a cheaper alternative (e.g. other non-medicinal plant species), either due to misidentification, or deliberately to cheat consumers, is an issue of growing concern. In this study, we used DNA barcoding to identify commonly used medicinal plants in South Africa. Using the core plant barcodes, *mat*K and *rbc*La, obtained from processed and poorly conserved materials sold at the muthi traditional medicine market, we tested efficacy of the barcodes in species discrimination. Based on genetic divergence, PCR amplification efficiency and BLAST algorithm, we revealed varied discriminatory potentials for the DNA barcodes. In general, the barcodes exhibited high discriminatory power, indicating their effectiveness in verifying the identity of the most common plant species traded in South African medicinal markets. BLAST algorithm successfully matched 61% of the queries against a reference database, suggesting that most of the information supplied by sellers at traditional medicinal markets in South Africa is correct. Our findings reinforce the utility of DNA barcoding technique in limiting false identification that can harm public health.

## Introduction

Traditional medicine is regarded as the most famous health care system in the world (WHO 2002), likely because of its accessibility and popularity. Currently, over 80% of human population around the globe relies on medicinal plants for their daily fight for better health (WHO 2002). In Africa, access to modern medical treatment is very limited largely due to lack of facilities or, when hospitals exist; their services are unaffordable for the majority. As a result, medicinal plants are extensively used to meet people’s needs for health care (Staden 1999, Hostettman et al. 2000, WHO 2002, Fyhrquist 2007, Koduru et al. 2007).

South Africa has a rich tropical and temperate flora, harbouring approximately 24 000 species, which account for more than 10% of the world’s vascular plants (Germishuizen and Meyer 2003). Of this unique diversity, approximately 3 000 species (~13%) are used as medicines, with a large number of them exported to other countries even outside Africa (Van Wyk et al. 1997).

In the recent past, harvesting medicinal plants was the domain of trained traditional healers, well known for their skills as herbalists or diviners who respected customary conservation practices (Cunningham 1993). Today, however, the gathering and trading of medicinal plants is no longer restricted to traditional healers but has entered informal commercial sectors of the South African economy, resulting in an increase in the number of herbal gatherers and traders (Dold and Cocks 2002). Mander (1998) recorded more than 100 000 traditional healers in South Africa. For example, in the Province of KwaZulu-Natal alone, between 20 000 and 30 000 people, mainly women, make their living from trade of non-timber forest products, particularly medicinal plants (Mander 1998). This intensive gathering of plants from the wild poses a serious threat to South Africa’s rich biodiversity (Dold and Cocks 2002), increases risk of extinction (Hoareau and DaSilva 1999) and leads to scarcity of commonly used medicinal plants (Cunningham 1991, Mander 1997, 1998, Dold and Cocks 2002). Species such as *Ocotea bullata* (Burch.) Baill., *Warburgia salutaris* (G. Bertol.) Chiov. and *Bowie volubilis* Harv. ex Hook. f., which were once abundant, are now threatened with extinction due to over-harvesting in the wild (www.redlist.sanbi.org). In addition, some species such as *Cassine transvaalensis* (Burtt Davy) Codd, and *Erythrophleum lasianthum* Corbishley, are now becoming threatened also due to over-harvesting in the wild (Fennel et al. 2004). Given the increasing pressure on medicinal plants, there is a need for increasing commitment towards efficient controls and better practices that can help preserve medicinal plant diversity in South Africa.

**Figure 1.**
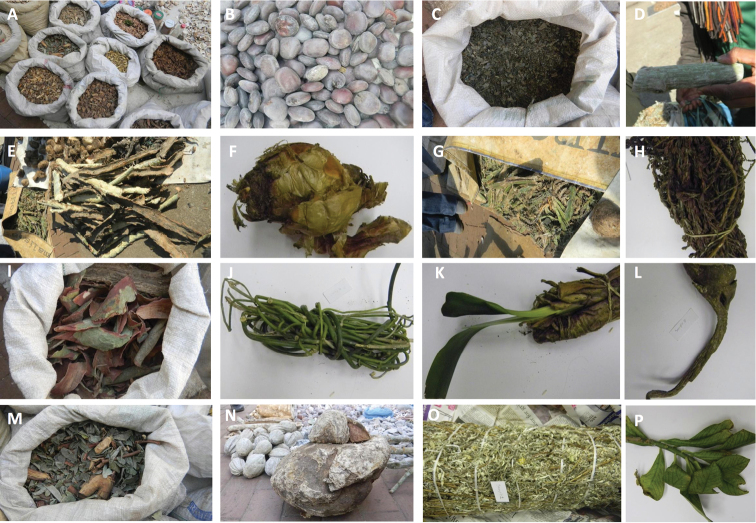
Examples ofmedicinal herbs bought at Faraday muthi market in Johannesburg **A** different medicinal herbs in bags **B** Seeds of *Entada rheedii* (tindili) **C** mixed herbs (fembo) **D** A twig of *Adenia gummifera* (mphinde umshaye) **E** Barks of *Vachellia* sp. (umkhanya-kute) **F** Bulb of *Boophane disticha* (umqotho) **G** mixed herbs **H** *Myrothamnus flabellifolius* (vuka) **I** Barks of *Vachellia* sp. (umkhanya-kute) **J** *Sarcostemma viminale* (ube nam) **K** Plant of *Clivia* sp. (mayime) **L** *Stangeria eriopus* (imfingo) **M** mixed herbs (isihlalakahle) **N** Tuber (umbonsi) **O** *Helichrysum* sp. (impepo) and **P** Twigs of *Synadenium cupulare* (umdletshane). Names in brackets are vernacular names in isiZulu.

To reach this objective, the primary step requires a reliable tool for accurate plant identification. Traditional plant identification is based on morphological characteristics, which can be problematic especially for medicinal plants that are mainly traded as dried or processed barks, dried leaves, roots, and stems (Figure 1) in popular markets known in South Africa as muthi market. As such, traded medicinal plants are devoid of identification diagnostics making morphologically-based identification non applicable (Dold and Cocks 2002). Also, medicinal plants cover a broad range of taxa, which may be phylogenetically less related but morphologically very similar. Such similarity between species may lead to misidentification and inappropriate use (Chen et al. 2010). This is of high concern as it may cause fatalities especially given that several medicinal plants are poisonous (Watt and Breyer-Brandwijk 1962, Van Wyk et al. 2002, Bruni et al. 2010). For instance, WHO (2004) reported in Hong Kong, fourteen cases of accidental substitution of the roots of *Gentiana* and *Clematis* species with that of *Podophyllum hexandrum* Royle for their antiviral qualities due to similarity in the morphological features of their roots. Similarly, a serious case of cardiac arrhythmias was reported as a side effect, caused by the accidental substitution of plantain (*Plantago major* L.; used as dietary supplements) with *Digitalis lanata* Ehrh. (used for heart conditions; WHO 2004). In the early 2000’s, large quantities of misidentified plantains were shipped to more than 150 manufacturers, distributors and retailers in the United States over a period of two years (WHO 2004). Another case of misidentification was in India, where mustard oil was accidentally contaminated with seeds of *Argemone mexicana* L., resulting in an epidemic of dropsy (WHO 2004). The misidentification of these seeds could have been avoided if there had been proper quality control of source materials (WHO 2004).

Given such alarming situations of misidentification, developing techniques to assist and support traditional plant identification (e.g. assigning dried barks, roots or leaves to species) is an urgent matter not only to preserve biodiversity and traditional knowledge attached to each plant (Yessoufou 2005) but also to secure human health (Chen et al. 2010). From this perspective, we propose that the use of DNA barcoding can assist in distinguishing species and assigning unidentified individuals or any plant organs or materials to species level (Kress et al. 2005, Kress and Erickson 2008, Lahaye et al. 2008, Kesanakurti et al. 2011). DNA barcoding is the use of a short gene sequence from a standardised region of the genome that could – in principle – distinguish between even closely related species (Hebert et al. 2004, Lahaye et al. 2008, Kesanakurti et al. 2011, Van der Bank et al. 2012). Ideally, DNA barcoding studies use fresh or well-preserved materials as sources of DNA. However, this is not always practical in many situations where DNA is already degraded because materials are either already processed or poorly preserved. Such situations include diet analyses (Huang 1972), ancient DNA studies (Pääbo et al. 2004), specimen identification from environmental DNA samples (Gratz 2004) and medicinal materials in muthi markets.

Two DNA regions were recently proposed as core barcodes, *rbc*La and *mat*K (CBOL 2009) with their identification efficacy estimated at 70-80% for land plants. The efficacy of DNA barcodes has rarely been evaluated for plant materials that are poorly stored or already processed; to our knowledge only one recent study has evaluated this with regards to animals where the discriminatory power of a mini-barcode was assessed in processed materials (Boyer et al. 2012). In this study, we focus on poorly conserved and processed medicinal plant materials sold in a South African muthi market with specific emphasis on commonly used plants. First, we constructed a DNA barcode library for these medicinal plants using fresh materials. Second, we bought poorly conserved and processed materials sold at the muthi market, and tested the efficacy of the core barcodes in assigning these processed materials to their species using the DNA barcode library as the reference.

## Material and methods

### Taxon sampling

A total of 108 species belonging to 55 plant families were identified as commonly used medicinal plants in South Africa based on a literature survey (Hutchings et al. 1996, Van Wyk et al. 1997, Van Wyk and Gericke 2000) (see Appendix). We collected these plants from several localities in four Provinces in South Africa: Gauteng, Limpopo, Mpumalanga, and the Western Cape. Our sampling comprised 185 specimens (see Appendix). Collection details, taxonomy, voucher numbers, GPS coordinates, field pictures, and sequence data (*mat*K and *rbc*La) are archived online on the Barcode of Life Data Systems (BOLD) (www.boldsystems.org). The voucher specimens for all the taxa as well as GenBank and BOLD accession numbers are listed in the Appendix.

In addition, we included in this study, plant materials bought from the Faraday muthi market (henceforth muthi samples) in Johannesburg, South Africa. A muthi market is a popular market where trade and services in African traditional medicines are provided to the general public. Materials sold in this market include various plant parts such as dried or fresh leaves, seeds, barks, and roots, etc. (Figure 1). These materials are sometimes in poorly stored and/or processed states (e.g. powder). In total, we included 18 additional muthi samples in our sampling and recorded their vernacular names (mainly in isiZulu) as provided by the sellers. It was not possible to assign scientific names to the samples at the time of purchase as they were in poor condition or had already been processed.

### DNA extraction, amplification, sequencing and alignment

Of the 108 species collected from the wild, leaf samples of 37 species were sent to the Canadian Centre for DNA Barcoding (CCDB) in Canada, where total DNA was extracted, the two core DNA barcodes (*mat*K and *rbc*La) were amplified and sequenced according to CCDB protocols. The sequencing for the remaining 71 species was done at the African Centre for DNA Barcoding (ACDB) in South Africa. The 18 muthi samples were also processed and sequenced at the ACDB.

DNA extraction followed the 2 × CTAB method (Doyle and Doyle 1987). Polyvinyl pyrolidone (2% PVP) was added to reduce the effect of high polysaccharide concentration in the samples. After precipitating the DNA with 100% ethanol, it was stored at -20 °C for a minimum of two weeks (Fay et al. 1998). DNA extracts were purified using QIAquik silica columns (Qiagen Inc., Hilden, Germany) according to the manufacturers’ protocol.

For both genes, PCR amplification was performed using ReadyMix Mastermix (Advanced Biotechnologies, Epson, Surrey, UK). We added 3.2% bovine serum albumin (BSA) to all reactions to serve as stabilizer for enzymes, to reduce problems with secondary structure, and improve annealing (Palumbi 1996). PCR amplification was performed using either the 9800 Fast Thermal Cycler or the GeneAmp PCR System 9700 machines. PCR programs used are as follows: (a) for *rbc*La, pre-melt at 94 °C for 60 s, denaturation at 94 °C for 60 s, annealing at 48 °C for 60 s, extension at 72 °C for 60 s (for 28 cycles), followed by a final extension at 72 °C for 7 min, and (b) for *mat*K, the protocol consisted of pre-melt at 94 °C for 3 min, denaturation at 94 °C for 60 s, annealing at 52 °C for 60 s, extension at 72 °C for 2 min (for 30 cycles), final extension at 72 °C for 7 min.

Cycle sequencing reactions were carried out in a GeneAmp PCR System 9700 thermal cycler using the ABI PRISM® BigDye® Terminator v3.1 (Applied Biosystems, Inc., California, USA). Cycle sequencing products were precipitated in ethanol and sodium acetate to remove excess dye terminators. Then suspended into 10 µL HiDi formamide (ABI) before sequencing on a ABI 3130 *xl* Genetic Analyzer (ABI).

Complementary strands were assembled and edited using Sequencher v3.1 (Gene Codes, Ann Arbor, Michigan, USA). All the sequences generated at ACDB and CCDB including those retrieved from BOLD were aligned manually in PAUP* v4.0b.10 (Swofford 2002).

### Data analyses

All analyses were conducted in the R package Spider (Brown et al. 2012). Only species for which sequences of both genes (*rbc*La and *mat*K) were available were included in the analyses. First, we evaluated K2P-interspecific and intraspecific genetic distances using Wilcoxon’s sum rank test and the significance of the differences between both distances was tested. Second, we determined the genetic distance suitable as threshold with which to test the efficacy of the DNA regions in assigning sequences to species. Third, we tested the identification efficacy used medicinal plants using three distance-based methods: best close match (Meier et al. 2006), near neighbour, and species identification methods used by BOLD (www.boldsystems.org). The best close match and near neighbour analyses measure the identification efficacy by searching for the closest individuals; the former focuses on a single nearest neighbour match, whereas the latter considers all matches within a specific threshold. The BOLD species identification method performed species delimitation based on a distance cut-off of 1%.

We then evaluated the ability of the core DNA barcodes in assigning poorly conserved or already-processed plant materials to species. For this test, the barcoding technique was applied on all 18 muthi samples. Our procedure here consisted of two steps. The first involved the use of vernacular names (in isiZulu) for the muthi samples to identify their scientific names based on Hutchings et al. (1996). The second step was based on the BLAST algorithm implemented in the BOLD identification system (www.boldsystems.org/index.php/IDS_OpenIdEngine) for *mat*K and *rbc*L sequences. The BLAST algorithm measures the efficiency of species identification against a global data repository such as BOLD or GenBank (Munch et al. 2008). The program takes a query of the sequence and matches it against the database selected by the user. The E-value and maximum identity are two statistics that can be used to measure the efficiency of species identification. The results are reported in a rank list whereby the closer the hit is to 100% and the E-value to 0, the better the identification efficiency. The DNA sequences generated from the 18 poorly conserved and degraded muthi samples were BLASTed against the reference database of medicinal plants available on the BOLD system. For additional evidence to the BLAST test, we included the sequences of muthi samples (as queries) in the database of DNA matrix generated for all medicinal plants, and reconstructed a maximum parsimony (MP) phylogeny based on the combined DNA matrix. Our objective here was to trace on the phylogeny, the positions of muthi samples (our queries) along the phylogenetic tree. Support for the groupings was analysed using bootstrapping. Maximum parsimony analysis was performed using PAUP* v4.0b10 (Swofford 2002). Tree searches were done using heuristic searches with 1 000 random sequence additions but keeping only 10 trees. Tree bisection-reconnection was performed with all character transformations treated as equally likely i.e. Fitch parsimony (Fitch 1971). Bootstrap resampling (Felsenstein 1985) was done also in PAUP* v4.0b10 (Swofford 2002). Node support was assessed based on the following scale: BS 50-74% (weak bootstrap support) and 75-100% for strong support (Hillis and Bull 1993, Murphy et al. 2001, Daru et al. 2013).

## Results

**Figure 2.**
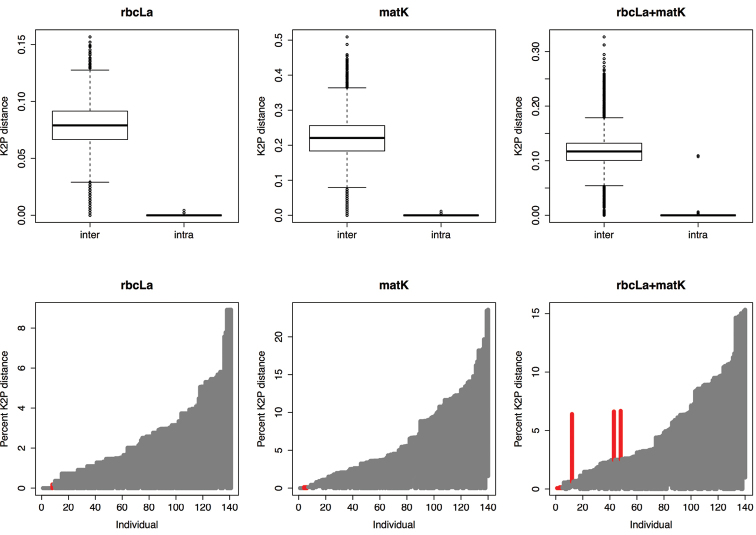
Evaluation of barcode gaps in *mat*K, *rbc*La and *rbc*La + *mat*K for commonly used medicinal plants of South Africa. **A** Boxplots indicate the genetic variation between interspecific distance and intraspecific distance; the boxplots clearly shows significant differences between inter- and intraspecific distances for all gene regions tested (P < 0.001; see text) **B** Lineplot of the barcode gap for the commonly used plants in South African medicine. For each gene region, the grey lines correspond to the furthest intraspecific distance (bottom of line value), and the closest interspecific distance (top of line value). The red lines show where this relationship is reversed, i.e. cases where there is no barcode gap.

Based on genetic divergence, *rbc*La exhibits the lowest mean interspecific distance (0.08); in contrast, *mat*K exhibits the highest mean interspecific distance, which almost doubles that of *rbc*La + *mat*K (0.22 versus 0.119 respectively). From the genetic variation test based on K2P-distance for *mat*K, we found that interspecific distance was significantly higher than intraspecific (inter_median_ = 0.232 vs. intra_median_ = 0.00; Wilcoxon sum rank test, p < 0.001; Table 1), indicating that a barcode gap exists for *mat*K. Also, a similar pattern was found for *rbc*La, high significant difference between inter- and intraspecific distances (inter_median_ = 0.07 vs. intra_median_ = 0.001, p < 0.001). We also found that when *rbc*La and *mat*K were combined the interspecific distance was significantly higher than intraspecific distance (inter_median_ = 0.12 vs. intra_median_ = 0.00, p < 0.001). Furthermore, our analyses indicate that a clear barcode gap exist between the range of intra- versus interspecific distances for all regions (Figure 2).

**Table 1.** Summary statistics indicating the range and means of intra- and interspecific distances for the gene regions and combination tested.

DNA regions	Numbers of sequences	Sequence length	K	Range inter	Mean inter (±SD)	Range intra	Mean intra (±SD)	Threshold (%)
*rbc*La	141	552	0.06	0–0.16	0.080±0.022	0–0.004	0.0002±0.0007	0.63
*mat*K	140	915	0.03	0–0.51	0.220±0.066	0–0.012	0.0008±0.0022	1.44
*rbc*La+*mat*K	140	1467	0.05	0–0.33	0.119±0.035	0–0.109	0.0039±0.0196	1.25

The Tajima’s K index of sequence was divergence measured as the mean number of substitutions per nucleotide which indicates that *mat*K had the lowest sequence divergence (3%) whereas *rbc*La and *rbc*La + *mat*K had similar divergence indices of 6% and 5% respectively.

We calculated the optimised genetic distance (threshold) with which the discriminatory power for different gene regions was evaluated. Apart from *rbc*La for which the optimised threshold was lower than 1%, both *mat*K and *rbc*La + *mat*K had optimised thresholds greater than 1% (i.e. 1.44% and 1.25% respectively). Using these cut-offs, we then evaluated the discriminatory power of different regions. We found that the combination *rbc*La + *mat*K provided the best discriminatory power based on the near neighbour and the best close match methods (96% and 97% respectively, Table 2). However, using the BOLD identification criteria, the discriminatory power of the combined regions dropped to 85% which is close to 86% for *mat*K alone but higher than that of *rbc*La (76%). Also, the application of BOLD identification criteria results in higher proportion of ambiguous identification: *rbc*La (23%), *mat*K (10%) and *rbc*La + *mat*K (11%). Conversely, the best close match method had the lowest proportion of ambiguous identification (i.e. 0–7%) for all regions tested.

**Table 2.** Efficacy of DNA barcodes in identification of commonly used medicinal plants in South Africa.

DNA regions	Near Neighbour		BOLD (1%)				Best close match			
	False (%)	True (%)	Ambiguous (%)	Correct (%)	Incorrect (%)	No ID (%)	Ambiguous (%)	Correct (%)	Incorrect (%)	No ID (%)
*rbc*La	5	95	23	76	0	1	6	93	1	0
*mat*K	7	93	10	86	1	3	4	92	1	3
*rbc*La+*mat*K	4	96	11	85	1	3	0	97	2	1

We then BLASTed (compared) the sequences for the 18 poorly conserved and degraded muthi samples against the BOLD identification system. Two muthi samples proved difficult to amplify whereas the amplification was successful for the 16 remaining muthi samples (Table 3). Of the 16 samples, the BLAST test was successful for 11 samples (61%), indicating that the scientific names recovered from BLAST test matched perfectly the scientific names expected based on vernacular names. However, we found mismatches for five samples. These results were also indicated on the MP phylogeny presented in Figure 3.

**Figure 3.**
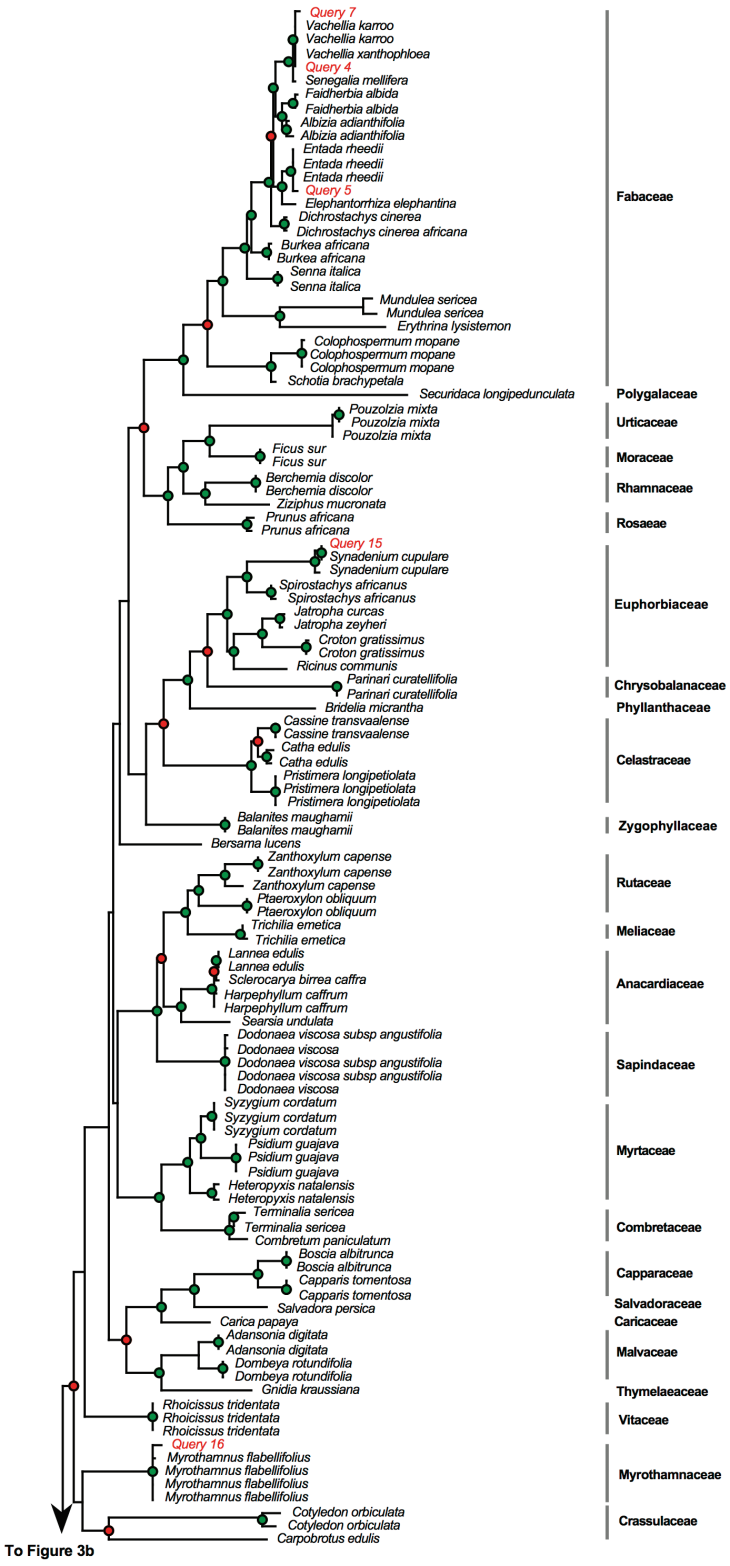
Phylogram obtained from the maximum parsimony analysis of matK with muthi samples included as “query”. Green dots indicate well-supported nodes (bootstrap support > 74%) and red dots indicate low bootstrap support (BS < 74%). Phylogram obtained from the maximum parsimony analysis of matK with muthi samples included as “query”. Green dots indicate well-supported nodes (bootstrap support > 74%) and red dots indicate low bootstrap support (BS < 74%).

**Figure 3.**
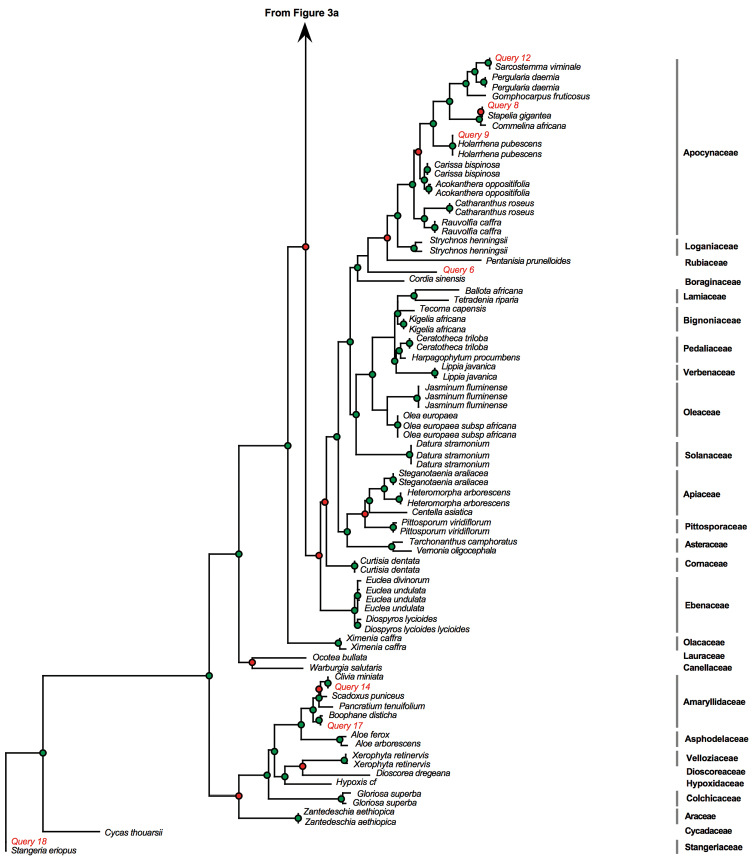
Continued.

**Table 3.** Comparison of BLAST results against common and scientific names for the muthi samples. **–** indicates specimens for which PCR failed. **?** indicates specimens for which common names or scientific names could not be found in the available literature. IUCN redlist obtained from http://redlist.sanbi.org

	Common names from “muthi” market	Common names from literature (in isiZulu; Hutchings et al. 2006)	Scientific names (Hutchings et al. 2006)	APG III (2009) Family	IUCN red list	BLAST sequence similarity - BOLD %	Do BLAST results match the correct scientific names?
1.	*impepo*	*impepo*	*Helichrysum* sp.	Asteraceae	-	100	True
2.	*isihlalakahle*	?	*Harworthia limifolia*	Asphodelaceae	Vulnerable (VU)	-	Amplification failed
3.	*fembo*	?	?	?	-	-	Amplification failed
4.	*mkhanya kute*	*umkhanya-kute*, *umdlovune*, *umhlofunga*, *umhlosinga*	*Vachellia xanthophloea*	Fabaceae	Least Concern (LC)	98	True
5.	*tindili*	*umbhone*, *tindili*	*Entada rheedii*	Fabaceae	Least Concern (LC)	99	True
6.	*ubhonsi*	?	*Mappia racemosa*	Icacinaceae	Vulnerable (VU)	89	False
7.	*ukhanyakute*	*umkhanya-kute*, *umdlovune*, *umhlofunga*, *umhlosinga*	*Vachellia xanthophloea*	Fabaceae	Least Concern (LC)	99	True
8.	*umlilo*	*uzililo*, *ililo elikhulu*	*Stapelia gigantea*	Apocynaceae	Least Concern (LC)	100	True
9.	*malilisa*	?	*Holarrhena pubescens*	Apocynaceae	Least Concern (LC)	100	False
10.	*umhlahlawehlathi*	*umhluswane*	*Cissus reniferus*	Vitaceae	-	97	False
11.	*mphinde umshaye*	?	*Adenia gummifera*	Passifloraceae	-	-	False
12.	*ube nam*	*umbelebele*, *umpelepe*	*Sarcostemma viminale*	Asclepiadaceae	Least Concern (LC)	100	False
13.	*isikholokhota*	*isikholokhoto*	*Sanseviera hyacinthiodes*	Asparagaceae	Least Concern (LC)	99	True
14.	*mayime*	*umayime*	*Clivia miniata*	Amaryllidaceae	Vulnerable (VU)	99	True
15.	*umdletshane*	*umbulele*, *umdletshane*	*Synadenium cupulare*	Euphorbiaceae	Least Concern (LC)	100	True
16.	*vuka*	*vuka*	*Myrothamnus flabellifolius*	Myrothamnaceae	DDT	100	True
17.	*umqotho*	*incotha*, *incwadi*	*Boophane disticha*	Amaryllidaceae	Declining	100	True
18.	*imfingo*	*imfingo*	*Stangeria eriopus*	Stangeriaceae	Vulnerable (VU)	100	True

## Discussion

The efficiency of a good barcode relies fundamentally on its ability to distinguish between closely related species. This is achieved only when there is enough genetic differentiation between rather than within species, i.e. when interspecific distance is significantly higher than intraspecific distance (Hebert et al. 2004, Savolainen et al. 2005, Lahaye et al. 2008). We tested this expectation on commonly used medicinal plants using *mat*K and *rbc*La. We found that both regions (*mat*K and *rbc*La) exhibit a significant barcode gap, suggesting that they should be efficient in assigning processed medicinal plants to species level. Further, the performance of each gene was very high for single and core barcodes (76–97%) but highest for the core under near neighbour and best close match methods. Overall, the core barcodes proves reliable in identifying commonly used medicinal plants of South Africa.

In several studies, the discriminatory power of the core barcodes has been questioned (Hollingsworth et al. 2009, Pettengill and Neel 2010, Roy et al. 2010, Wang et al. 2010, Clement and Donoghue 2012, Liu et al. 2012). These studies mainly focused on closely related species or single lineages. A recent study with a similar objective to ours also discounts the potential of the core barcodes in discriminating Chinese medicinal plants (Chen et al. 2010). The authors found a more reliable discriminatory power of 92.7% for ITS2 at the genus and species level from different plant families and closely related taxa. In our study, we did not include ITS2, but we found a similar power of 85% to 96% for the core barcodes (*mat*K and *rbc*La) in the context of South African commonly used medicinal plants. Chen et al. (2010) included 400 samples belonging to 326 species in 98 families covering dicots, monocots, gymnosperms and ferns of Chinese medicinal plants. Such broad sampling likely increased the probability of high proportion of closely related species, resulting in the low performance of the core barcodes in their study. However, our sampling size is limited to only commonly used medicinal plants (~108 species), and this restriction likely increases the chance of having less related species, leading to a higher performance we found for the core barcodes.

We further tested, the performance of the core barcodes by evaluating their identification efficacy on 18 medicinal plant products bought at the Faraday muthi market in Johannesburg, South Africa. The sequences generated from these 18 plant materials were BLASTed against the reference library on BOLD database system. Given that the plant materials sold at the muthi market were poorly conserved (dried, processed, etc.), we expected a very low percentage of DNA recovery and amplification. Possible explanation for the five samples that yielded false identification, and the two that failed are that the samples could be a mixture of leaves from multiple species. Such limitation could be overcome using individual sequencing of all components of mixed DNA samples based on high throughput sequencing techniques e.g. pyrosequencing technology, which is capable of simultaneously detecting many thousands of different sequences in a mixed sample, without the need for sub-cloning (Margulies et al. 2005).

Another possibility for the amplification failure observed in our study for some samples could be attributable to a bad post harvest condition of preservation, which may result in DNA degradation. Again, such limitation could be overcome through the search of a ‘mini-barcode’ (Meusnier et al. 2008, Boyer et al. 2012). The technique of sliding window analysis is now available for that purpose and has been proven reliable (Boyer et al. 2012). Given that medicinal plants are often poorly conserved or processed materials, the chance of successful extraction and amplification of long DNA fragments (> 200 bp) is very low (Meusnier et al. 2008, Boyer et al. 2012). As such, a search for shorter and informative fragment is necessary if we are to verify the identity of commonly used medicinal plants which are generally devoid of morphological features. Furthermore, we found some mismatch in species identification by the BLAST algorithm and the corresponding species based on vernacular names. Although, South African medicinal plants are well documented (e.g. Hutchings et al. 1996, Van Wyk et al. 1997), it remains highly likely that the mismatch might not be an artefact of erroneous claims from plant sellers, but presumably due to the variation of names used for the same plants across different ethnic groups.

The continual removal of medicinal plants from the wild has become worrisome in southern Africa (Setshogo and Mbereki 2011). Therefore, understanding the scarcity and popularity of plants at the muthi market is the starting point for conservation and evaluating threatened species (Williams et al. 2000, Setshogo and Mbereki 2011). For instance, Williams et al. (2000) mentioned *Helichrysum* sp. as being scarce and threatened in the future because of its popularity and demand at the muthi markets. The harvesting of the whole plant, bulb, tuber or roots before the seeds germinate damages the plant more than harvesting only leaves, seeds, bark or fruits (as seen in Figure 1). Although only about 22% of the muthi samples are currently threatened with extinction (Table 3), continual over-exploitation in the wild might eventually change the status for currently non-threatened species to threatened category. Therefore, there is an urgent need to conserve medicinal plants by cultivating them at home gardens (Setshogo and Mbereki 2011).

In conclusion, our analyses indicate that most of the information supplied by the sellers at the muthi market were correct. This could be due to the fact that we tested only 18 samples. Therefore, it remains possible that if we increase our sample size, we might detect important mismatch between the sellers’ claims and the products sold. We also propose a continued effort to increase the barcode library of South African medicinal plants, and in case of difficulties due to degraded materials, a pyro-sequencing technique in tandem with mini-barcodes is necessary. Our suggestions and findings are expected to be of great use in limiting false identification that can harm public health.

## Appendix

List of taxa with voucher information. (doi: 10.3897/zookeys.365.5730.app) File format: Microsoft Word file (docx).

**Explanation note:** List of taxa with voucher information, BOLD and GenBank accession numbers for each DNA region. English common names have been chosen. In cases where English common names were unavailable, names in native languages were used.

## References

[B1] Angiosperm Phylogeny Group III (2009) An update of APG classification for the orders and families of flowering plants. Botanical Journal of Linnean Society 161: 105-121. doi: 10.1111/j.1095-8339.2009.00996.x

[B2] Boyer S Brown SD Collins RA Cruickshank RH Lefort M Malumbres-Olarte J Wratten SD (2012) Sliding window analyses for optimal selection of mini-barcodes and application to 454-pyrosequencing for specimen identification from degraded DNA. PLoS ONE 7: e38215. doi: 10.1371/journal.pone.0038215 22666489 PMC3362555

[B3] Brown SDJ Collins RA Boyer S Lefort MC Malumbres-Olarte J Vink CJ Cruickshank RH (2012) Spider: An R package for the analysis of species identity and evolution, with particular reference to DNA barcoding. Molecular Ecology Resources 12: 562-565. doi: 10.1111/j.1755-0998.2011.03108.x 22243808

[B4] Bruni I De Mattia F Galimberti A Galasso G Banfi E Casiraghi M Labra M (2010) Identification of poisonous plants by DNA barcoding approach. International Journal of Legal Medicine 124: 595-603. doi: 10.1007/s00414-010-0447-3 20354712

[B5] CBOL Plant Working Group (2009) A DNA Barcode for land plants. Proceedings of the National Academy of Sciences of the USA 106: 12794-12797. doi: 10.1073/pnas.0905845106 19666622 PMC2722355

[B6] Chen S Yao H Han J Liu C Song J Shi L Zhu Y Ma X Gao T Pang X Luo P Li Y Li X Jia X Lin Y Leon C (2010) Validation of the ITS2 region as a novel DNA barcode for identifying medicinal plant species. PLoS ONE 5: e8613. doi: 10.1371/journal.pone.0008613 20062805 PMC2799520

[B7] Clement WL Donoghue MJ (2012) Barcoding success as a function of phylogenetic relatedness in *Viburnum*, a clade of woody angiosperms. BMC Evolutionary Biology 12: 73. doi: 10.1186/1471-2148-12-73 22646220 PMC3433329

[B8] Cunningham AB (1991) The herbal medicine trade: Resource depletion and environmental management for a hidden economy. In: Preston-whyte E Rogerson C (Eds) South Africa informal economy, chap. 12. Oxford University Press, Cape Town, 196-206.

[B9] Cunningham AB (1993) African medicinal plants: setting priorities at the interface between conservation and primary health care. People and plants working paper 1. UNESCO, Paris http://unesdoc.unesco.org/images/0009/000967/096707e.pdf

[B10] Daru BH Manning JC Boatwright JS Maurin O Maclean N Schaefer H Kuzmina M Van der Bank M (2013) Molecular and morphological analysis of subfamily Alooideae (Asphodelaceae) and the inclusion of *Chortolirion* in *Aloe*. Taxon 62: 62–76. http://www.ingentaconnect.com/content/iapt/tax/2013/00000062/00000001/art00006

[B11] Dold AP Cocks ML (2002) The trade in medicinal plants in the Eastern Cape Province, South Africa. South African Journal of Science 98: 589-597.

[B12] Doyle JJ Doyle JL (1987) A rapid DNA isolation procedure for small quantities of fresh leaf tissue. Phytochemical Bulletin 19: 11-15.

[B13] Fay MF Bayer C Alverson WS De Bruijn A Chase MW (1998) Plastid *rbc*L sequence data indicate a close affinity between *Diegodendron* and *Bixa*. Taxon 47: 43-50. doi: 10.2307/1224017

[B14] Felsenstein J (1985) Confidence levels on phylogenies: an approach using the bootstrap. Evolution 39: 783-791. doi: 10.2307/2408678 28561359

[B15] Fennel CW Light ME Sparg GI Stafford GI Van Staden J (2004) Assessing African medicinal plants for efficacy and safety: agricultural and storage practices. Journal of Ethnopharmacology 95: 113-121. doi: 10.1016/j.jep.2004.05.025 15507322

[B16] Fitch WM (1971) Towards defining the course of evolution: minimum change for a specified tree topology. Systematic Zoology 20: 406-416. doi: 10.1093/sysbio/20.4.406

[B17] Fyhrquist A (2007) Traditional medicinal uses and biological activities of some plant extracts of Africa *Combretum* Loefl., *Terminalia* L. and *Pteleopsis* Engl. species (Combretaceae). PhD thesis, Yliopistopaino, Helsinki.

[B18] Germishuizen G Meyer NL (2003) Plants of southern Africa: An annotated checklist. Strelitzia 14 National Botanical Institute, Pretoria.

[B19] Gratz NG (2004) Critical review of the vector status of *Aedes albopictus*. Medical and Veterinary Entomology 18: 215-227. doi: 10.1111/j.0269-283X.2004.00513.x 15347388

[B20] Hebert PDN Penton EH Burns JM Janzen DH Hallwachs W (2004) Ten species in one: DNA barcoding reveals cryptic species in the neotropical skipper butterfly *Astraptes fulgerator*. Proceedings of the National Academy of Sciences of the USA 101: 14812-14817. doi: 10.1073/pnas.0406166101 15465915 PMC522015

[B21] Hillis DM Bull JJ (1993) An empirical test of bootstrapping as a method for assessing confidence in phylogenetic analysis. Systematic Biology 42: 182-192. doi: 10.1093/sysbio/42.2.182

[B22] Hoareau L DaSilva EJ (1999) Medicinal plants: a re-emerging health aid. Journal of Biotechnology 2: 717–3458. http://www.scielo.cl/pdf/ejb/v2n2/art02.pdf

[B23] Hollingsworth ML Clark A Forrest LL Richardson J Pennington RT Long DG Cowan R Chase MW Gaudeul M Hollingsworth PM (2009) Selecting barcoding loci for plants: evaluation of seven candidate loci with species-level sampling in three divergent groups of land plants. Molecular Ecology Resources 9: 439-457. doi: 10.1111/j.1755-0998.2008.02439.x 21564673

[B24] Hostettman K Marston A Ndjoko K Wolfender JL (2000) The potential of African plants as a source of drugs. Current Organic Chemistry 4: 973-1010. doi: 10.2174/1385272003375923

[B25] Huang YM (1972) Contributions to the mosquito fauna of Southeast Asia. XIV. The subgenus *Stegomyia* of *Aedes* in Southeast Asia. I- The *Scutellaris* group of species. Contributions of the American Entomological Institute 9: 1–109. http://oai.dtic.mil/oai/oai?verb=getRecord&metadataPrefix=html&identifier=ADA510169

[B26] Hutchings A Scott AH Lewis G Cunnignham AB (1996) Zulu medicinal plants. University of Natal Press, Scottsville, South Africa.

[B27] Kesanakurti PR Fazekas AJ Burgess KS Percy DM Newmaster SG Graham SW (2011) Spatial patterns of plant diversity below ground as revealed by DNA barcoding. Molecular Ecology 20: 1289-1302. doi: 10.1111/j.1365-294X.2010.04989.x 21255172

[B28] Koduru S Grierson DS Afolayan AJ (2007) Ethnobotanical information of medicinal plants used for the treatment of cancer in the Eastern Cape province, South Africa. Current Science 92: 906-908.

[B29] Kress WJ Erickson DL (2008) A two-locus global DNA barcode for land plants: The coding *rbc*L gene complements the non-coding *trnH-psbA* spacer region. PLoS ONE 2: e508. doi: 10.1371/journal.pone.0000508 PMC1876818 17551588

[B30] Kress WJ Wurdack KJ Zimmer EA Weight IA Jazen DH (2005) Use of DNA barcodes to identify flowering plants. Proceedings of the National Academy of Sciences of the USA 102: 8369-8374. doi: 10.1073/pnas.0503123102 15928076 PMC1142120

[B31] Lahaye R Van der Bank M Bogarin D Warner J Pupulin F Gigot G Maurin O Duthoit S Barraclough TG Savolainen V (2008) DNA barcoding the floras of biodiversity hotspot. Proceedings of the National Academy of Sciences of the USA 105: 2923-2928. doi: 10.1073/pnas.0709936105 18258745 PMC2268561

[B32] Liu C Shi L Xu X Li H Xing H Liang D Jiang K Pang X Song J Chen S (2012) DNA barcode goes two-dimensions: DNA QR code web server. PLoS ONE 7: e35146. doi: 10.1371/journal.pone.0035146 22574113 PMC3344831

[B33] Mander M (1997) Medicinal plant marketing in Bushbuckridge and Mpumalanga: A market survey and recommended strategies for sustaining the supply of plants in the region. Unpublished report, Danish Cooperation for Environment and Development, Danish Environment Protection Agency, Strandgade.

[B34] Mander M (1998) Marketing of indigenous plants in South Africa. A case study in KwaZulu-Natal. Food and Agriculture Organization, Rome. http://www.fao.org/docrep/w9195e/w9195e00.htm

[B35] Margulies M Egholm M Ahman WE Attiya S Bader JS Bemben LA Berka J Braverman MS Chen YJ Chen Z Dewell SB Du L Fierro JM Gomes XV Godwin BC He W Helgesen S Ho CH Irzyk GP Jando SC Alenquer ML Jarvie TP Jirage KB Kim JB Knight JR Lanza JR Leamon JH Lefkowitz SM Lei M Li J Lohman KL Lu H Makhijani VB McDade KE McKenna MP Myers EW Nickerson E Nobile JR Plant R Puc BP Ronan MT Roth GT Sarkis GJ Simons JF Simpson JW Srinivasan M Tartaro KR Tomasz A Vogt KA Volkmer GA Wang SH Wang Y Weiner MP Yu P Begley RF Rothberg JM (2005) Genome sequencing in microfabricated high-density picolitre reactor. Nature 437: 376-380. doi: 10.1038/nature03959 16056220 PMC1464427

[B36] Meier R Shiyang K Vaidya G Ng PKL (2006) DNA barcoding and taxonomy in Diptera: a tale of high intraspecific variability and low identification success. Systematic Biology 55: 715–728. doi: 10.1080/10635150600969864 17060194

[B37] Meusnier I Singer GAC Landry J Hickey DA Hebert PDN Hajibabaei M (2008) A universal DNA mini-barcode for biodiversity analysis. BMC Genomics 9: 214. doi: 10.1186/1471-2164-9-214 18474098 PMC2396642

[B38] Munch K Boomsma W Huelsenbeck JP Willerslev E Nielsen R (2008) Statistical signment of DNA sequences using Bayesian phylogenetics. Systematic Biology 57: 750-757. doi: 10.1080/10635150802422316 18853361

[B39] Murphy WJ Eizirik E O’Brien SJ Madsen O Scally M Douady CJ Teeling E Ryder OA Stanhope MJ de Jong WW Springer MS (2001) Resolution of the early placental mammal radiation using Bayesian phylogenetics. Science 294: 2348-2351. doi: 10.1126/science.1067179 11743200

[B40] Pääbo S Poinar H Serre D Jaenicke-Despres V Hebler J Rohland N Kuch M Krause J Vigilant L Hofreiter M. (2004) Genetic analyses from ancient DNA. Annual Review of Genetics 38: 645-79. doi: 10.1146/annurev.genet.37.110801.143214 15568989

[B41] Palumbi SR (1996) Nucleic acids II: the polymerase chain reaction. In: Hillis DM Moritz C Mable BK Molecular Systematics, Second Edition Sinauer & Associates Inc. Publishers, Sunderland, 241–246.

[B42] Pettengill JB Neel MC (2010) An evaluation of candidate plant DNA barcodes and assignment methods in diagnosing 29 species in the genus *Agalinis* (Orobanchaceae). American Journal of Botany 97: 1381-1406. doi: 10.3732/ajb.0900176 21616891

[B43] Roy S Tyagi A Shulka V Kumar A Singh UM Chaudhary LB Datt B Bag SK Singh PK Nair NK Husain T Tuli R (2010) Universal plant DNA barcode loci may not work in complex groups: a case study with Indian *Berberis* species. PLoS ONE 5: e13674. doi: 10.1371/journal.pone.0013674 21060687 PMC2965122

[B44] Savolainen V Cowan RS Vogler AP Roderick GK Lane R (2005) Towards writing the encyclopedia of life: an introduction to DNA barcoding. Philosophical Transactions of the Royal Society 360: 1805-1811. doi: 10.1098/rstb.2005.1730 PMC1609222 16214739

[B45] Setshogo MP Mbereki CM (2011) Floristic diversity and uses of medicinal plants sold by street vendors in Gaborone, Botswana. The African Journal of Plant Science and Biotechnology 5(1): 69-74.

[B46] Staden JV (1999) Medicinal plants in southern Africa: utilization, sustainability, conservation – can we change mindsets? Outlook on Agriculture 28: 75–76.

[B47] Swofford DL (2002) PAUP*. Phylogenetic Analysis Using Parsimony (*and Other Methods). 4b10 Ed Sinauer Associates, Sunderland, Massachusetts.

[B48] Van der Bank HF Greenfield R Daru BH Yessoufou K (2012) DNA barcoding reveals micro-evolutionary changes and river system-level phylogeographic resolution of seven populations of African silver catfish, *Schilbe intermedius* (Siluriformes, Schilbeidae). Acta Ichthyologica et Piscatoria 42: 307-320. doi: 10.3750/AIP2012.42.4.04

[B49] Van Wyk B-E Gericke N (2000) People’s plants: A guide to useful plants of southern Africa. Briza Publications, Pretoria.

[B50] Van Wyk B-E Van Heerden F Van Oudtshoorn B (2002) Poisonous plants of South Africa. Briza Publications, Pretoria, South Africa.

[B51] Van Wyk B-E Van Oudtshoorn B Gericke N (1997) First Edition. Medicinal plants of South Africa. Briza Publications, Pretoria.

[B52] Wang W Wu Y Yan Y Ermakova M Kerstetter R Messing J (2010) DNA barcoding of the Lemnaceae, a family of aquatic monocots. BMC Plant Biology 10: 205. doi: 10.1186/1471-2229-10-205 20846439 PMC2956554

[B53] Watt JM Breyer-Brandwijk MG (1962) Second Edition. The Medicinal and Poisonous plants of Southern and Eastern Africa. Livingstone, London.

[B54] Williams VL Balkwill K Witkowski ETF (2000) Unraveling the commercial market for medicinal plants and plant parts on the Witwatersrand, South Africa. Economic Botany 54: 310-327. doi: 10.1007/BF02864784

[B55] World Health Organization (2002) WHO launches the first global strategy on traditional and alternative medicine. Geneva, Switzerland http://www.who.int/mediacentre/news/releases/release38/en/12528386

[B56] World Health Organization (2004) Medicinal plants – guidelines to promote patient safety and plant conservation for a US$ 60 billion industry. http://www.who.int/mediacentre/news/notes/2004/np3/en/

[B57] Yessoufou K (2005) Ecological and ethnobotanical research on *Irvingia gabonensis* and *Blighia sapida* in Plateau Province, Eastern Benin. MSc thesis, University of Abomey-Calavi, Benin.

