# Supplementary material for: Efficacy of the core DNA barcodes in identifying processed and poorly conserved plant materials commonly used in South African traditional medicine
Source: Zookeys. 2013 Dec 30;(365):215–33. doi: 10.3897/zookeys.365.5730 (PMC3890679; doi:10.3897/zookeys.365.5730)
Supplement: Supplementary file 1 — List of taxa with voucher information. (doi: 10.3897/zookeys.365.5730.app) File format: Microsoft Word file (docx). [file ZooKeys-365-215-s001.docx]

| **APG III Family** | **Full Botanical Name** | **Common Name** | **Voucher / Herbarium** | **Accession Number** | | |
| --- | --- | --- | --- | --- | --- | --- |
|  |  |  |  |  |  |  |
|  |  |  |  |  |  |  |
|  |  |  |  | **BOLD** | **GenBank** | |
|  |  |  |  |  | ***matK*** | ***rbcL*** |
| Aizoaceae | *Carpobrotus edulis* (L.) N.E.Br. | sour fig | *LTM0017*/JRAU | SAFH2063-11 | JQ024942 | JQ025027 |
| Amaryllidaceae | *Boophane disticha* Herb. | bushman poison bulb | *OM0730*/JRAU | SAFH840-10 | JQ024938 | JQ025023 |
| Amaryllidaceae | *Clivia miniata* (Lindl.) Bosse | bush lily, orange lily | *LTM0022*/JRAU | SAFH2542-11 | JQ024949 | JQ025034 |
| Amaryllidaceae | *Pancratium tenuifolium* Hochst. ex A.Rich. | dwaalboom | *OM1606*/JRAU | SAFH634-10 | JQ024979 | JQ025067 |
| Amaryllidaceae | *Scadoxus puniceus* (L.) Friis & Nordal | red paint brush | *OM1643*/JRAU | SAFH641-10 | JQ024994 | JQ025086 |
| Anacardiaceae | *Harpephyllum caffrum* Bernh. ex C.Krauss | wild plum | *OM1555*/JRAU | KNPA957-09 | **JF270814** | **JF265467** |
| Anacardiaceae | *Harpephyllum caffrum* Bernh. ex C.Krauss | wild plum | *BS0058*/JRAU | SAFH1954-11 | **JQ412249** | **JQ412371** |
| Anacardiaceae | *Lannea edulis* (Sond.) Engl. | wild grape | *OM1991*/JRAU | KNPA1116-09 | − | − |
| Anacardiaceae | *Lannea edulis* (Sond.) Engl. | wild grape | *OM1971*/JRAU | KNPA1096-09 | **JF270844** | **JF265497** |
| Anacardiaceae | *Sclerocarya birrea* subsp. *caffra* (Sond.) Kokwaro | marula | *OM0498*/JRAU | KNPA1266-09 | **JF270929** | **JF265586** |
| Anacardiaceae | *Searsia undulata* (Jacq.) T.S.Yi, A.J.Mill. & J.Wen | kuni bush | *OM2940*/JRAU | SAFH2152-11 | JQ024996 | JQ025088 |
| Apiaceae | *Centella asiatica* (L.) Urb. | penny wort | *LTM030*/JRAU | SAFH2073-11 | JQ024945 | JQ025030 |
| Apiaceae | *Heteromorpha arborescens* (Spreng.) Cham. & Schltdl. | parsley tree | *OM592*/JRAU | KNPA1283-09 | − | − |
| Apiaceae | *Heteromorpha arborescens* (Spreng.) Cham. & Schltdl. | parsley tree | *OM2726*/JRAU | SAFH1647-11 | − | − |
| Apiaceae | *Steganotaenia araliacea* Hochst. | carrot tree | *OM2540*/JRAU | SAFH1461-11 | − | − |
| Apiaceae | *Steganotaenia araliacea* Hochst. | carrot tree | *OM1350*/JRAU | KNPA893-09 | JQ25001 | JQ025095 |
| Apocynaceae | *Acokanthera oppositifolia* (Lam.) Codd | bushman's poison bush | *OM1782*/JRAU | KNPA032-08 | **JF270622** | **JF265265** |
| Apocynaceae | *Acokanthera oppositifolia* (Lam.) Codd | bushman's poison bush | *OM3240*/JRAU | SAFH2371-11 | − | − |
| Apocynaceae | *Carissa bispinosa* (L.) Desf. ex Brenan | common num num | *AM0296*/JRAU | SAFH1947-11 | − | − |
| Apocynaceae | *Carissa bispinosa* (L.) Desf. ex Brenan | common num num | *OM3229*/JRAU | SAFH2361-11 | − | − |
| Apocynaceae | *Catharanthus roseus* (L.) G.Don | madagascar periwinkle | *OM1613*/JRAU | SAFH637-10 | JQ024944 | JQ025029 |
| Apocynaceae | *Catharanthus roseus* (L.) G.Don | madagascar periwinkle | *BS0161*/JRAU | SAFH2034-11 | **JQ412220** | **JQ412337** |
| Apocynaceae | *Gomphocarpus fruticosus* (L.) W.T.Aiton | milk weed | *LTM0001*/JRAU | SAFH2056-11 | − | − |
| Apocynaceae | *Holarrhena pubescens* Wall. | fever pod | *OM1926*/JRAU | KNPA1069-09 | **JF270823** | **JF265476** |
| Apocynaceae | *Holarrhena pubescens* Wall. | fever pod | *OM2083*/JRAU | SAFH239-10 | − | − |
| Apocynaceae | *Pergularia daemia* (Forssk.) Chiov. | trellis vine | *OM0743*/JRAU | SAFH846-10 | − | − |
| Apocynaceae | *Pergularia daemia* (Forssk.) Chiov. | trellis vine | *OM1244*/JRAU | SAFH538-10 | − | − |
| Apocynaceae | *Rauvolfia caffra* Sond. | quinine tree | *OM1376*/JRAU | KNPA898-09 | JQ024990 | JQ025082 |
| Apocynaceae | *Rauvolfia caffra* Sond. | quinine tree | *RBN0216*/JRAU | KNPA1384-09 | **JF270912** | **JF265569** |
| Apocynaceae | *Sarcostemma viminale* (L.) R.Br. | melktou | *OM1649*/JRAU | SAFH644-10 | JQ024993 | JQ025085 |
| Apocynaceae | *Stapelia gigantea* N.E. Br. | giant carrion flower | *OM1349*/JRAU | SAFH569-10 | JQ25000 | JQ025094 |
| Araceae | *Zantedeschia aethiopica* (L.) Spreng. | arum lily | *BS0072*/JRAU | SAFH2517-11 | **JQ412304** | **JQ412438** |
| Araceae | *Zantedeschia aethiopica* (L.) Spreng. | arum lily | *LTM0018*/JRAU | SAFH2540-11 | JQ025015 | JQ025108 |
| Asphodelaceae | *Aloe arborescens* Mill. | krantz aloe | *Abbott9167*/JRAU | SAFH390-10 | − | − |
| Asphodelaceae | *Aloe ferox* Mill. | bitter aloe | *Abbott9235*/JRAU | SAFH456-10 | − | − |
| Asteraceae | *Tarchonanthus camphoratus* L. | wild camphor bush | *OM1515*/JRAU | KNPA936-09 | JQ025005 | JQ025099 |
| Asteraceae | *Vernonia oligocephala* (DC.) Sch.Bip. ex Walp. | groenamara | *OM0587*/JRAU | KNPA1282-09 | JQ025011 | JQ025104 |
| Bignoniaceae | *Kigelia africana* (Lam.) Benth. | sausage tree | *OM0217*/JRAU | KNPA1166-09 | **JF270837** | **JF265490** |
| Bignoniaceae | *Kigelia africana* (Lam.) Benth. | sausage tree | *OM3497*/JRAU | SAFH3485-11 | − | − |
| Bignoniaceae | *Tecoma capensis* (Thunb.) Lindl. | cape honeysuckle | *OM0454*/JRAU | TSA199-10 | − | − |
| Boraginaceae | *Cordia sinensis* Lam. | omusepa | *OM0354*/JRAU | KNPA1242-09 | **JF270723** | **JF265370** |
| Canellaceae | *Warburgia salutaris* (G.Bertol.) Chiov. | pepper bark tree | *OM1853*/JRAU | KNPA088-08 | **JF270994** | **JF265653** |
| Capparaceae | *Boscia albitrunca* (Burch.) Gilg & Benedict | sherpherds tree | *OM0312*/JRAU | KNPA1222-09 | **JF270660** | **JF265307** |
| Capparaceae | *Boscia albitrunca* (Burch.) Gilg & Benedict | sherpherds tree | *OM1274*/JRAU | SAFH547-10 | − | − |
| Capparaceae | *Capparis tomentosa* Lam. | wooly caper bush | *RL1155*/JRAU | KNPA1433-09 | − | − |
| Capparaceae | *Capparis tomentosa* Lam. | wooly caper bush | *OM1112*/JRAU | SAFH507-10 | − | − |
| Caricaceae | *Carica papaya* L. | pawpaw tree | *LTM0027*/JRAU | SAFH2070-11 | JQ024941 | JQ025026 |
| Celastraceae | *Cassine transvaalensis* (Burtt Davy) Codd | transvaal saffronwood | *OM0403*/JRAU | KNPA1252-09 | **JF270757** | **JF265407** |
| Celastraceae | *Cassine transvaalensis* (Burtt Davy) Codd | transvaal saffronwood | *OM1229*/JRAU | SAFH534-10 | − | − |
| Celastraceae | *Catha edulis* (Vahl) Endl. | bushman's tea | *RL1155*/JRAU | − | − | − |
| Celastraceae | *Catha edulis* (Vahl) Endl. | bushman's tea | *BS0153*/JRAU | SAFH2026-11 | **JQ412219** | **JQ412336** |
| Celastraceae | *Pristimera longipetiolata* (Oliv.) N. Hallé | chipanga | *OM0227B*/JRAU | KNPA1173-09 | JQ024984 | JQ025074 |
| Celastraceae | *Pristimera longipetiolata* (Oliv.) N. Hallé | chipanga | *OM1098*/JRAU | KNPA819-09 | − | − |
| Celastraceae | *Pristimera longipetiolata* (Oliv.) N. Hallé | chipanga | *OM0393*/JRAU | KNPA549-09 | **JF270901** | **JF265558** |
| Chrysobalanaceae | *Parinari curatellifolia* Planch. ex Benth. | mobola plum | *OM2621*/JRAU | SAFH1542-11 | − | − |
| Chrysobalanaceae | *Parinari curatellifolia* Planch. ex Benth. | mobola plum | *OM0281*/JRAU | KNPA1207-09 | − | − |
| Colchicaceae | *Gloriosa superba* L. | flame lily | *OM0633*/JRAU | SAFH807-10 | − | − |
| Colchicaceae | *Gloriosa superba* L. | flame lily | *OM0375*/JRAU | SAFH717-10 | − | − |
| Combretaceae | *Combretum paniculatum* Vent. | flame creeper | *RL1661*/JRAU | KNPA650-09 | JQ024950 | JQ025035 |
| Combretaceae | *Terminalia sericea* Burch. ex DC. | silver cluster leaf | *OM1037.1*/JRAU | KNPA799-09 | − | − |
| Combretaceae | *Terminalia sericea* Burch. ex DC. | silver cluster leaf | *OM0478*/JRAU | KNPA554-09 | **JF270968** | **JF265626** |
| Commelinaceae | *Commelina africana* L. | yellow commelina | *OM342A*/JRAU | SAFH703-10 | JQ024951 | JQ025036 |
| Cornaceae | *Curtisia dentata* (Burm.f.) C.A.Sm. | assegai | *OM1737*/JRAU | − | − | − |
| Cornaceae | *Curtisia dentata* (Burm.f.) C.A.Sm. | assegai | *OM3167*/JRAU | SAFH2310-11 | − | − |
| Crassulaceae | *Cotyledon orbiculata* L. | pigs ear | *LTM0014*/JRAU | SAFH2062-11 | JQ024952 | JQ025037 |
| Crassulaceae | *Cotyledon orbiculata* L. | pigs ear | *BS0129*/JRAU | SAFH2009-11 | **JQ412227** | **JQ412346** |
| Cycadaceae | *Cycas thouarsii* R. Br. ex Gaudich. | madagascar cycad | *Little & Stevenson 1001* (NY) | − | **AF394336** | **AB116589** |
| Dioscoreaceae | *Dioscorea dregeana* (Kunth) T.Durand & Schinz | wild yam | *OM1465*/JRAU | SAFH602-10 | JQ024957 | JQ025042 |
| Ebenaceae | *Diospyros lycioides* Desf. | bluebush | *OM0965*/JRAU | KNPA1330-09 | **JF270741** | **JF265389** |
| Ebenaceae | *Diospyros lycioides* Desf. | bluebush | *OM2126*/JRAU | SAFH282-10 | − | − |
| Ebenaceae | *Euclea divinorum* Hiern | magic guarri | *OM1102*/JRAU | SAFH503-10 | **JF270767** | **JF265418** |
| Ebenaceae | *Euclea undulata* Thunb. | common guarri | *OM1572*/JRAU | SAFH628-10 | JQ024962 | JQ025046 |
| Ebenaceae | *Euclea undulata* Thunb. | common guarri | *OM2939*/JRAU | SAFH2151-11 | − | − |
| Ebenaceae | *Euclea undulata* Thunb. | common guarri | *OM3058*/JRAU | SAFH2237-11 | − | − |
| Euphorbiaceae | *Croton gratissimus* Burch. | lavender croton | *OM1946*/JRAU | SAFH655-10 | − | − |
| Euphorbiaceae | *Croton gratissimus* Burch. | lavender croton | *OM0547*/JRAU | KNPA556-09 | − | − |
| Euphorbiaceae | *Jatropha curcas* L. | purging nut | *OM1182*/JRAU | SAFH521-10 | − | − |
| Euphorbiaceae | *Jatropha zeyheri* Sond. | verfbol | *OM0416*/JRAU | TSA118-10 | JQ024972 | JQ025058 |
| Euphorbiaceae | *Ricinus communis* L. | castor oil plant | *OM1359*/JRAU | KNPA895-09 | **JF270918** | **JF265575** |
| Euphorbiaceae | *Spirostachys africana* Sond. | tamboti | *OM0990*/JRAU | KNPA1338-09 | JQ024999 | JQ025092 |
| Euphorbiaceae | *Spirostachys africana* Sond. | tamboti | *OM0254*/JRAU | KNPA1191-09 | **JF270944** | **JF265602** |
| Euphorbiaceae | *Synadenium cupulare* L.C. Wheeler | dead man's tree | *OM1511*/JRAU | KNPA935-09 | JQ025004 | JQ025098 |
| Euphorbiaceae | *Synadenium cupulare* L.C. Wheeler | dead man's tree | *OM0757*/JRAU | KNPA568-09 | **JF270958** | **JF265616** |
| Fabaceae | *Albizia adianthifolia* (Schum.) W.Wight | flat crown | *OM2159*/JRAU | SAFH312-10 | JQ024935 | JQ025020 |
| Fabaceae | *Albizia adianthifolia* (Schum.) W.Wight | flat crown | *OM2610*/JRAU | SAFH1531-11 | − | − |
| Fabaceae | *Burkea africana* Hook. | wild seringa | *OM1953*/JRAU | KNPA1086-09 | JQ024939 | JQ025024 |
| Fabaceae | *Burkea africana* Hook. | wild seringa | *OM2128*/JRAU | SAFH284-10 | − | − |
| Fabaceae | *Colophospermum mopane* (Benth.) Leonard | mopane | *OM0778*/JRAU | KNPA1304-09 | **JF270696** | **JF265343** |
| Fabaceae | *Colophospermum mopane* (Benth.) Leonard | mopane | *RL1558*/JRAU | SAFH1035-10 | − | − |
| Fabaceae | *Dichrostachys cinerea* subsp. *africana* Brenan & Brummitt | sickle bush | *RBN0359*/JRAU | KNPA578-09 | **JF270739** | **JF265387** |
| Fabaceae | *Dichrostachys cinerea* subsp. *africana* Brenan & Brummitt | sickle bush | *OM0256*/JRAU | KNPA1192-09 | JQ024956 | JQ025041 |
| Fabaceae | *Elephantorrhiza elephantina* (Burch.) Skeels | elandsbean | *OM0483*/JRAU | KNPA123-08 | **JF270759** | **JF265409** |
| Fabaceae | *Entada rheedii* Spreng. | sea bean | *OM2417*/JRAU | TSA242-10 | JQ024960 | JQ025045 |
| Fabaceae | *Entada rheedii* Spreng. | sea bean | *OM2670*/JRAU | SAFH1591-11 | − | − |
| Fabaceae | *Entada rheedii* Spreng. | sea bean | *OM3508*/JRAU | SAFH3496-11 | − | − |
| Fabaceae | *Erythrina lysistemon* Hutch. | common coral tree | *RBN0329*/JRAU | KNPA1411-09 | **JF270764** | **JF265415** |
| Fabaceae | *Faidherbia albida* (Delile) A.Chev. | ana tree | *OM3495*/JRAU | SAFH3483-11 | − | − |
| Fabaceae | *Faidherbia albida* (Delile) A.Chev. | ana tree | *RBN165.1*/JRAU | KNPA1364-09 | **JF270778** | **JF265429** |
| Fabaceae | *Mundulea sericea* (Willd.) A.Chev. | silver bush | *OM0993*/JRAU | KNPA1339-09 | − | − |
| Fabaceae | *Mundulea sericea* (Willd.) A.Chev. | silver bush | *OM2625*/JRAU | SAFH1546-11 | − | − |
| Fabaceae | *Mundulea sericea* (Willd.) A.Chev. | silver bush | *RL1572*/JRAU | − | JQ024974 | JQ025062 |
| Fabaceae | *Schotia brachypetala* Sond. | weeping boer bean | *OM1166*/JRAU | KNPA836-09 | JQ024995 | JQ025087 |
| Fabaceae | *Senegalia mellifera* (Vahl) Seigler & Ebinger subsp. *mellifera* | black thorn | *OM1030*/JRAU | KNPA795-09 | JQ024932 | JQ025017 |
| Fabaceae | *Senna italica* Mill. | wild senna | OM0417/JRAU | SAFH736-10 | JQ024997 | JQ025090 |
| Fabaceae | *Senna italica* Mill. | wild senna | *OM0309*/JRAU | SAFH699-10 | − | − |
| Fabaceae | *Vachellia karroo* (Hayne) Banfi & Galasso | sweet thorn | *RL1282.1*/JRAU | KNPA1453-09 | **JF270609** | **JF265252** |
| Fabaceae | *Vachellia karroo* (Hayne) Banfi & Galasso | sweet thorn | *OM3013*/JRAU | SAFH2205-11 | − | − |
| Fabaceae | *Vachellia* *xanthophloea* (Benth.) P.J.H.Hurter | fever tree | *OM2579*/JRAU | SAFH1500-11 | − | − |
| Hypoxidaceae | *Hypoxis* cf. L. | *−* | *OM0548*/JRAU | ALOAF378-10 | JQ024969 | JQ025056 |
| Lamiaceae | *Ballota africana* (L.) Benth. | kattekruie | *OM3057*/JRAU | SAFH2236-11 | − | − |
| Lamiaceae | *Tetradenia riparia* (Hochst.) Codd | ginger bush | *OM0881*/JRAU | KNPA350-09 | **JF270969** | **JF265627** |
| Lauraceae | *Ocotea bullata* (Burch.) Baill. | black stinkwood | *Abbott9194*/JRAU | SAFH417-10 | JQ024978 | JQ025066 |
| Loganiaceae | *Strychnos henningsii* Gilg | red bitterberry | *OM2381*/JRAU | TSA163-10 | JQ025002 | JQ025096 |
| Loganiaceae | *Strychnos henningsii* Gilg | red bitterberry | *Abbott9223*/JRAU | SAFH444-10 | − | − |
| Malvaceae | *Adansonia digitata* L. | boabab | *OM0747*/JRAU | KNPA1297-09 | **JF270625** | **JF265268** |
| Malvaceae | *Adansonia digitata* L. | boabab | *OM1306*/JRAU | KNPA885-09 | JQ024933 | JQ025018 |
| Malvaceae | *Dombeya rotundifolia* Planch. | wild pear | *OM1120*/JRAU | KNPA826-09 | − | − |
| Malvaceae | *Dombeya rotundifolia* Planch. | wild pear | *OM0489*/JRAU | TSA202-10 | JQ024959 | JQ025044 |
| Meliaceae | *Ekebergia capensis* Sparrm. | cape ash | *OM1540*/JRAU | KNPA949-09 | **JF270756** | − |
| Meliaceae | *Trichilia emetica* Vahl | natal mahogonay | *OM2103*/JRAU | SAFH259-10 | JQ025007 | JQ025100 |
| Meliaceae | *Trichilia emetica* Vahl | natal mahogonay | *OM1178*/JRAU | KNPA840-09 | **JF270977** | **JF265636** |
| Melianthaceae | *Bersama lucens* (Hochst.) Szyszyl. | glossy bersama | *OM1562*/JRAU | KNPA959-09 | **JF270657** | **JF265304** |
| Moraceae | *Ficus sur* Forssk. | broom cluster fig | *OM1556*/JRAU | KNPA102-08 | **JF270786** | **JF265438** |
| Moraceae | *Ficus sur* Forssk. | broom cluster fig | *OM2135*/JRAU | SAFH291-10 | − | − |
| Myrothamnaceae | *Myrothamnus flabellifolius* Welw. | ressurection plant | *OM1209*/JRAU | KNPA851-09 | − | − |
| Myrothamnaceae | *Myrothamnus flabellifolius* Welw. | ressurection plant | *OM1137*/JRAU | SAFH511-10 | JQ024976 | JQ025064 |
| Myrothamnaceae | *Myrothamnus flabellifolius* Welw. | ressurection plant | *OM0285*/JRAU | KNPA1210-09 | − | − |
| Myrothamnaceae | *Myrothamnus flabellifolius* Welw. | ressurection plant | *OM3332*/JRAU | SAFH3320-11 | − | − |
| Myrtaceae | *Heteropyxis natalensis* Harv. | lavender tree | *OM1407*/JRAU | KNPA906-09 | JQ024968 | JQ025055 |
| Myrtaceae | *Heteropyxis natalensis* Harv. | lavender tree | *OM1944*/JRAU | TSA326-10 | − | − |
| Myrtaceae | *Psidium guajava* L. | guava | *CS0036*/JRAU | KNPA698-09 | JQ024986 | JQ025077 |
| Myrtaceae | *Psidium guajava* L. | guava | *LTM0026*/JRAU | SAFH2069-11 | JQ024987 | JQ025078 |
| Myrtaceae | *Psidium guajava* L. | guava | *JG0049*/JRAU | SAFH2991-11 | − | − |
| Myrtaceae | *Syzygium cordata* Hochst ex C. Krauss | water berry | *RBN0304*/JRAU | KNPA1408-09 | **JF270959** | **JF265617** |
| Myrtaceae | *Syzygium cordata* Hochst ex C. Krauss | water berry | *OM0951*/JRAU | KNPA187-08 | − | − |
| Myrtaceae | *Syzygium cordata* Hochst ex C. Krauss | water berry | *OM1470*/JRAU | SAFH605-10 | − | − |
| Olacaceae | *Ximenia caffra* Sond. | sourplum | *OM2056*/JRAU | SAFH212-10 | JQ025013 | JQ025106 |
| Olacaceae | *Ximenia caffra* Sond. | sourplum | *RL1182*/JRAU | KNPA111-08 | − | − |
| Oleaceae | *Jasminum fluminense* Vell. | okarondo | *OM0273*/JRAU | KNPA1201-09 | JQ024970 | JQ025057 |
| Oleaceae | *Jasminum fluminense* Vell. | okarondo | *RL1254*/JRAU | KNPA099-08 | − | − |
| Oleaceae | *Jasminum fluminense* Vell. | okarondo | *OM0456*/JRAU | KNPA1258-09 | **JF270833** | **JF265486** |
| Oleaceae | *Olea europaea* L. subsp. *africana* (Mill.) P.S.Green | wild olive | *OM2818*/JRAU | SAFH1698-11 | − | − |
| Oleaceae | *Olea europaea* L. subsp. *africana* (Mill.) P.S.Green | wild olive | *OM0269*/JRAU | KNPA1198-09 | **JF270876** | **JF265533** |
| Oleaceae | *Olea europaea* L. subsp. *africana* (Mill.) P.S.Green | wild olive | *BS0123*/JRAU | SAFH2005-11 | **JQ412273** | **JQ412400** |
| Pedaliaceae | *Ceratotheca triloba* (Bernh.) Hook.f. | wild foxglove | *OM0651*/JRAU | KNPA1290-09 | JQ024946 | JQ025031 |
| Pedaliaceae | *Ceratotheca triloba* (Bernh.) Hook.f. | wild foxglove | *OM1367*/JRAU | SAFH3209-11 | − | − |
| Pedaliaceae | *Harpagophytum procumbens* (Burch.) DC. ex Meisn. | devil's claws | *OM0477*/JRAU | TSA201-10 | JQ025052 | JQ024966 |
| Phyllanthaceae | *Bridelia micrantha* (Hochst.) Baill. | mitzeeri | *OM1435*/JRAU | KNPA510-09 | **JF270668** | **JF265315** |
| Pittosporaceae | *Pittosporum viridiflorum* Sims | cheese wood | *OM1738*/JRAU | KNPA152-08 | JQ024982 | JQ025072 |
| Pittosporaceae | *Pittosporum viridiflorum* Sims | cheese wood | *OM2815*/JRAU | SAFH1697-11 | − | − |
| Polygalaceae | *Securidaca longipedunculata* Fresen. | violet tree | *OM3358*/JRAU | SAFH3346-11 | − | − |
| Rhamnaceae | *Berchemia discolor* (Klotzsch) Hemsl. | brown ivory | *OM1175*/JRAU | KNPA838-09 | **JF270655** | **JF265302** |
| Rhamnaceae | *Berchemia discolor* (Klotzsch) Hemsl. | brown ivory | *OM2437*/JRAU | TSA262-10 | − | − |
| Rhamnaceae | *Ziziphus mucronata* Willd. | buffalo thorn | *OM0258*/JRAU | KNPA185-08 | **JF271007** | **JF265666** |
| Rosaceae | *Prunus africana* (Hook. f.) Kalkman | fever pod | *OM1568*/JRAU | KNPA139-08 | JQ024985 | JQ025076 |
| Rosaceae | *Prunus africana* (Hook. f.) Kalkman | fever pod | *YM0002*/JRAU | − | − | − |
| Rubiaceae | *Pentanisia prunelloides* (Klotzsch) Walp. | wild verbena | *OM0612*/JRAU | SAFH795-10 | JQ024980 | JQ025070 |
| Rutaceae | *Ptaeroxylon obliquum* (Thunb.) Radlk. | sneeze wood | *OM1326*/JRAU | KNPA890-09 | JQ024988 | JQ025079 |
| Rutaceae | *Ptaeroxylon obliquum* (Thunb.) Radlk. | sneeze wood | *OM0815*/JRAU | KNPA142-08 | **JF270904** | **JF265562** |
| Rutaceae | *Zanthoxylum capense* (Thunb.) Harv. | small knob wood | *OM1775*/JRAU | KNPA027-08 | **JF271004** | **JF265663** |
| Rutaceae | *Zanthoxylum capense* (Thunb.) Harv. | small knob wood | *OM3231*/JRAU | SAFH2362-11 | − | − |
| Salvadoraceae | *Salvadora persica* L. | mustard seed | *OM0824*/JRAU | KNPA178-08 | **JF270925** | **JF265582** |
| Sapindaceae | *Dodonaea viscosa* Jacq. | hopbush | *Abbott9229*/JRAU | SAFH450-10 | − | − |
| Sapindaceae | *Dodonaea viscosa* Jacq. | hopbush | *OM2689*/JRAU | SAFH1610-11 | − | − |
| Sapindaceae | *Dodonaea viscosa* subsp. *angustifolia* (L.f.) J.G.West | sand olive | *OM2129*/JRAU | SAFH285-10 | − | − |
| Sapindaceae | *Dodonaea viscosa* subsp. *angustifolia* (L.f.) J.G.West | sand olive | *OM2918*/JRAU | SAFH2133-11 | JQ024958 | JQ025043 |
| Sapindaceae | *Dodonaea viscosa* subsp. *angustifolia* (L.f.) J.G.West | sand olive | *PG0002*/JRAU | SAFH2415-11 | − | − |
| Solanaceae | *Datura stramonium* L. | thornapple | 1001 (NY) | SAFH1977-11 | **JQ412230** | **JQ412350** |
| Solanaceae | *Datura stramonium* L. | thornapple | *OM3431*/JRAU | SAFH3419-11 | − | − |
| Solanaceae | *Datura stramonium* L. | thornapple | *PR0936*/JRAU | SAFH3643-11 | − | − |
| Stangeriaceae | *Stangeria eriopus* (Kunze) Baill. | stangeria | *PR0706*/JRAU | CYAF057-10 | − | JQ025707 |
| Thymeleaceae | *Gnidia kraussiana* Meisn. | yellow heads | *Mol.Sys.0010*/JRAU | TSA322-10 | JQ024965 | JQ025050 |
| Urticaceae | *Pouzolzia mixta* Solms | soap nettle | *RL1276*/JRAU | KNPA1450-09 | − | − |
| Urticaceae | *Pouzolzia mixta* Solms | soap nettle | *OM572*/JRAU | KNPA180-08 | **JF270899** | **JF265556** |
| Urticaceae | *Pouzolzia mixta* Solms | soap nettle | *OM3328*/JRAU | SAFH3316-11 | − | − |
| Velloziaceae | *Xerophyta retinervis* Baker | monkey's tail | *OM1591*/JRAU | KNPA969-09 | JQ025013 | JQ025106 |
| Velloziaceae | *Xerophyta retinervis* Baker | monkey's tail | *OM1213*/JRAU | SAFH3204-11 | − | − |
| Verbenaceae | *Lippia javanica* (Burm.f.) Spreng. | fever tea | *OM0215*/JRAU | KNPA1164-09 | **JF270850** | **JF265503** |
| Verbenaceae | *Lippia javanica* (Burm.f.) Spreng. | fever tea | *RBN0348*/JRAU | TSA227-10 | − | − |
| Vitaceae | *Rhoicissus tridentata* (L. f.) Wild & R.B. Drumm. | wild grape | *OM0452*/JRAU | TSA198-10 | JQ024991 | JQ025083 |
| Vitaceae | *Rhoicissus tridentata* (L. f.) Wild & R.B. Drumm. | wild grape | *RL1130*/JRAU | SAFH700-10 | − | − |
| Vitaceae | *Rhoicissus tridentata* (L. f.) Wild & R.B. Drumm. | wild grape | *OM0249*/JRAU | KNPA1188-09 | **JF270917** | **JF265574** |
| Zygophyllaceae | *Balanites maughamii* Sprague | torchwood | *OM0223*/JRAU | KNPA1170-09 | **JF270650** | **JF265296** |
| Zygophyllaceae | *Balanites maughamii* Sprague | torchwood | *OM0994*/JRAU | KNPA1340-09 | − | − |
